# Ginsenoside Rg1 Alleviates Podocyte EMT Passage by Regulating AKT/GSK3 *β*/*β*-Catenin Pathway by Restoring Autophagic Activity

**DOI:** 10.1155/2020/1903627

**Published:** 2020-01-30

**Authors:** Yimin Shi, Yanbin Gao, Tao Wang, Xiaolei Wang, Jiaxin He, Jiayi Xu, Bingjie Wu, Yimeng Li

**Affiliations:** ^1^Department of Endocrinology, School of Traditional Chinese Medicine, Capital Medical University, Beijing, China; ^2^Department of Endocrinology, Beijing Key Lab of Traditional Chinese Medicine Collateral Disease Theory Research, Capital Medical University, Beijing, China

## Abstract

**Background:**

Diabetic nephropathy (DN), a complication of diabetes, is the result of high glucose-induced pathological changes in podocytes, such as epithelial-mesenchymal transition (EMT). Autophagy is an important mechanism of podocyte repair. Ginsenoside Rg1, the active ingredient of ginseng extract, has antifibrotic and proautophagic effects. Therefore, we hypothesized that ginsenoside Rg1 can reverse podocyte EMT via autophagy and alleviate DN.

**Aim:**

This study aimed to investigate the effect of ginsenoside Rg1 on DN rats and high glucose-induced podocyte EMT by regulating the AKT/GSK3*β*/*β*/

**Methods:**

Diabetic rats induced by STZ injection were treated with 50 mg/kg ginsenoside Rg1 for 8 weeks, and the renal functional, metabolic, and histopathological indices were evaluated. DN was simulated *in vitro* by exposing podocytes to high glucose levels and treated with ginsenoside Rg1. The expression of EMT and autophagy-related markers was analyzed *in vivo* and *in vitro* by exposing podocytes to high glucose levels and treated with ginsenoside Rg1. The expression of EMT and autophagy-related markers was analyzed

**Results:**

Ginsenoside Rg1 significantly alleviated renal fibrosis and podocyte EMT in diabetic rats, and podocytes exposed to high glucose levels, which was abolished by the autophagy inhibitor 3-MA. Furthermore, ginsenoside Rg1 regulated the AKT/GSK3 *β*/*β*/

**Conclusion:**

Ginsenoside Rg1 alleviated podocyte EMT by enhancing AKT/GSK3*β*/*β*-catenin pathway-mediated autophagy, indicating its therapeutic potential for DN and other glomerular diseases.*β*/*β*/

## 1. Introduction

Diabetic nephropathy (DN) is a serious microvascular complication of diabetes [1] and a common cause of chronic renal failure. The prominent pathological feature of DN is glomerular sclerosis, which manifests as proteinuria in the early stages of the disease, and is an important indicator of its severity [2]. The mechanistic basis of proteinuria in DN is podocyte injury, which is the result of apoptosis and the subsequent decrease in their number and density [3, 4]. However, recent studies show that the number of glomerular podocytes does not change significantly prior to or during microalbuminuria in diabetic rats [5]. An alternative possibility is high glucose-induced epithelial-mesenchymal transition (EMT) of the podocytes, which disrupts their structure and function, and damages the integrity of the glomerular filtration barrier leading to proteinuria, glomerular sclerosis, and renal dysfunction. EMT of the mature podocytes is accompanied by the loss of epithelial markers like nephrin and concomitant increase in mesenchymal markers like *α*-smooth muscle actin (SMA) [6, 7].

Autophagy has gained considerable attention in recent years as the possible pathological basis of DN [8]. It is a highly conserved intracellular catabolic process which recycles damaged organelles and maintains cell homeostasis and metabolism during stress conditions [9]. Since podocytes are terminally differentiated cells with poor proliferative capacity, they need a higher degree of autophagy even under physiological conditions [10]. To date, more than 30 autophagy-related proteins (Atg) have been identified, which are necessary for autophagy and its related processes [11]. Autophagy begins with the formation of the autophagosome and the induction of the beclin-1 (Atg6) complex, which also consists of the class III phosphoinositide 3 kinase (PI3K) hVps34, which docks at the site of autophagosome initiation. The first of the two ubiquitin-like systems involved in autophagy binds to the ubiquitin-like proteins Atg12 to Atg5, which then binds to Atg16L1 to form the second ubiquitin-like system. This complex binds to and activates Atg3 and Atg7, which recruits LC3-I to the autophagosomes. Lipidation of LC3-I with phosphatidyl ethanolamine (PE) forms LC3-II, which closes the autophagosomes around specific cargos and adaptor proteins like p62 [12,13]. The exact role of podocyte autophagy in EMT and the pathogenesis of DN is still unclear.

Ginseng has been used in traditional Chinese medicine formulations for more than 2,000 years and has shown anti-transdifferentiation, antioxidant, antiapoptotic, and autophagic effects [14–16]. The pharmacological effects of ginseng are mainly attributed to the ginsenosides, primarily the ginsenoside Rg1. The aim of this study was to determine its effects on podocyte autophagy and EMT following hyperglycemic injury in both *in vitro* and *in vivo* models of DN.

## 2. Materials and Methods

### 2.1. Reagents

**Figure 1:**
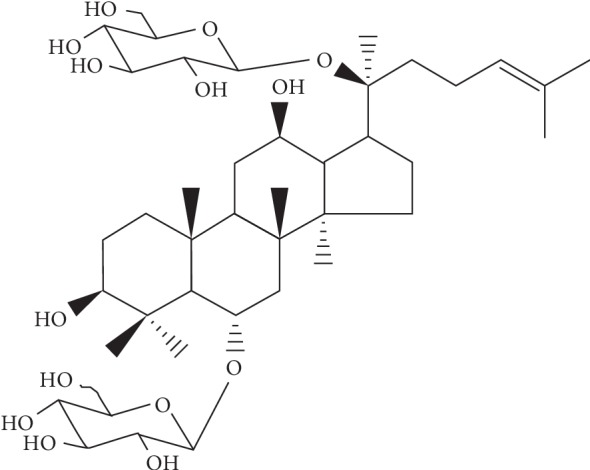
Chemical structure of Ginsenoside Rg1.

Ginsenoside Rg1 (Figure 1, C_42_H_72_O_14_, molecular weight = 801.01, purity by high-performance liquid chromatography (HPLC) ≥ 98%) was purchased from Solarbio. Rapamycin and 3-MA were bought from Selleck Chemicals and STZ from Sigma.

### 2.2. Establishment of Murine DN Model and Treatment

SPF-grade male Sprague-Dawley rats (aged 8 weeks, weighing 180–200 g) were purchased from the Beijing Vital River Laboratory Animal Technology Co. Ltd. The animals were housed in the Laboratory Animal Center of Capital Medical University at 24 ± 1°C and a 12 h light/dark cycle. All experiments were conducted in accordance with the guidelines for the care and use of laboratory animals of the National Institutes of Health and approved by the Animal Welfare Committee of the Animal Laboratory of Capital Medical University.

Diabetes was induced by intraperitoneally injecting the rats with 50 mg/kg STZ (streptozocin), and 8 rats were injected with an equal volume of the vehicle (0.1 M citrate buffer, pH 4.5) as the placebo/normal control (NC, *n* = 8). Three days after injection, tail vein blood was collected and serum glucose was measured, and fasting glucose level ≥16.7 mM for 3 consecutive days was indicative of diabetes. The diabetic rats were fed with high-fat diet (HFD; 10% lard, 20% sucrose, 2.5% cholesterol, 0.5% sodium cholate, and 67% basic feed), while the control rats were given normal food. Four weeks after STZ injection, the diabetic rats were randomly divided into the (untreated) DN group (*n* = 8) and ginsenoside Rg1-treated group (*n* = 8). The latter was administered 50 mg/kg ginsenoside Rg1 once daily by intraperitoneal injection for 8 weeks, and the other groups received the vehicle for the same duration. At the end of the regimen, urine creatinine (UCr), urinary microalbumin (Malb), blood urea nitrogen (BUN), and serum creatinine (SCr) were measured. The rats were sacrificed, and their renal cortices were collected for further analyses.

### 2.3. Cell Culture

The conditionally immortalized mouse podocyte line MPC5 was purchased from the National Platform of Experimental Cell Resources for Sci-Tech. The cells were cultured at 33°C in DMEM/low glucose medium supplemented with 10% fetal bovine serum (FBS) and recombinant IFN-*γ*. To induce differentiation of the podocytes, they were grown at 37°C without recombinant IFN-*γ* in the same medium and then in serum-free conditions for 24 h once they reached 80% confluency. The differentiated podocytes were cultured under the following conditions: normal glucose (normal group, DMEM containing 5.5 mM glucose), normal glucose containing mannitol (mannitol group, DMEM containing 5.5 mM glucose and 24.5 mM mannitol), high glucose (HG group, DMEM containing 5.5 mM glucose and 24.5 mM glucose), and high glucose with ginsenoside Rg1 (Rg1 group, DMEM containing 5.5 mM glucose and 24.5 mM glucose and 40 *μ*g/ml ginsenoside Rg1) for 48 h.

### 2.4. Histological Examination

Kidney tissues were fixed in 4% paraformaldehyde, embedded in paraffin, and sliced into 3 *μ*m thick sections. After dewaxing with xylene and rehydrating through an alcohol gradient, the sections were stained with hematoxylin and eosin, periodic acid Schiff (PAS), and Masson's stain according to standard protocols. For electron microscopy, the cortex tissues were immobilized with 2% glutaraldehyde in 0.1 M phosphate buffer at 4°C for 2 h. After gradient dehydration, the tissues were osmotically embedded and cut into ultrathin sections that were then double stained with 4% uranyl acetate and lead citrate.

### 2.5. Western Blotting

The podocytes and kidney tissues were lysed, and equivalent amount of protein (20 g) per sample was separated by 10% SDS-PAGE and transferred to PVDF membranes. After blocking with 5% skimmed milk in the TBST buffer, the membranes were incubated with antibodies against nephrin, *α*-smooth muscle actin (*α*-SMA), beclin-1, p62, AKT, P-Akt, GSK3*β*, p-GSK3*β* (all from Abcam, UK), and LC3-II (Sigma). The blots were washed and incubated with the HRP-conjugated secondary antibody and developed using chemiluminescence reagents.

### 2.6. Real-Time RT-PCR

Total RNA was isolated from the cells/tissues using TRIzol® reagent according to the manufacturer's instructions and reverse transcribed using the SuperScript RT kit. The SYBR Green kit was used for qRT-PCR, and the 2ΔCT method was used to calculate the relative gene expression levels. The sequence of primers is shown in Table 1.

### 2.7. Immunofluorescence

The tissue sections were processed as described above and, after antigen retrieval, blocked with sheep serum for 1 hour. The sections were incubated overnight at 4°C with the primary antibodies. After counterstaining with DAPI for 5 minutes, the sections were observed under a fluorescence microscope.

### 2.8. Statistical Analysis

SPSS software (IBM, USA) was used for statistical analysis. All data were expressed as the average (±SD) of at least three independent experiments, and analyzed by single factor variance. *P* < 0.05 was considered statistically significant.

## 3. Results

### 3.1. Ginsenoside Rg1 Improved Renal Function and Tissue Architecture in DN Rats

**Figure 2:**
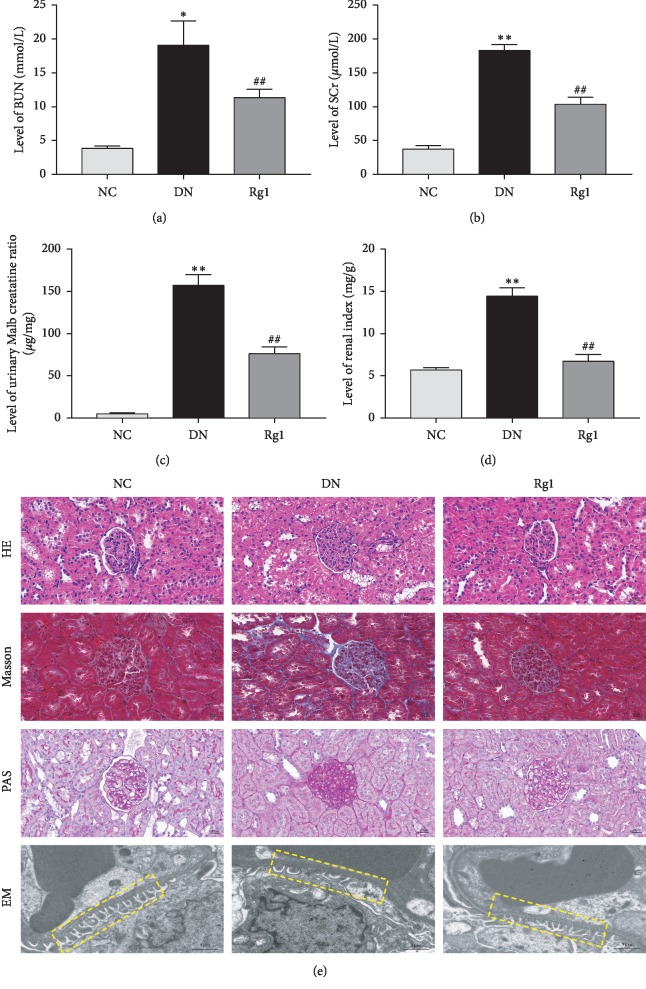
Effect of ginsenoside Rg1 on renal function in DN SD rats: (a) BUN; (b) SCr; (c) urinary Malb creatinine ratio; (d) renal index; (e) representative photograph for HE, Masson, PAS staining; EM, representative images of GBM thickening and podocyte morphology; *n* = 8. ^*∗*^*P* < 0.05 and ^*∗∗*^*P* < 0.01 as compared with the NC group; ^#^*P* < 0.05 and ^##^*P*  <  0.01 as compared with the DN group. Abbreviations: BUN, blood urea nitrogen; SCr, serum creatinine; Malb, microalbuminuria; HE, hematoxylin-eosin; PAS, periodic acid Schiff; EM, electron microscopy; GBM, glomerular basement membrane.

Compared to the control animals, all indices of renal function-renal weight/body weight ratio and the levels of serum creatinine, urea nitrogen, urinary creatinine, and urinary microalbumin were significantly increased in the DN group. Ginsenoside Rg1 improved the above parameters in the DN rats (see Figures 2(a)–2(d)), indicating an ameliorative effect on renal metabolism and proteinuria. Histologically, the renal cortex of the DN rats showed obvious glomerular hypertrophy with diffuse and nodular sclerosis, excessive glycogen storage (see Figure 2(e)), and collagen deposition in the glomeruli (see Figure 2(e)). In addition, electron microscopy examination showed a loose and irregularly arranged glomerular basement membrane (GBM), with podocyte fusion, rupture, and loss (see Figure 2(e)). Treatment with ginsenoside Rg1 significantly improved the pathological changes and restored the glomerular structure. Taken together, ginsenoside Rg1 had a significant therapeutic effect on DN rats by improving the metabolic and histopathological indices.

### 3.2. Ginsenoside Rg1 Inhibited Podocyte EMT

**Figure 3:**
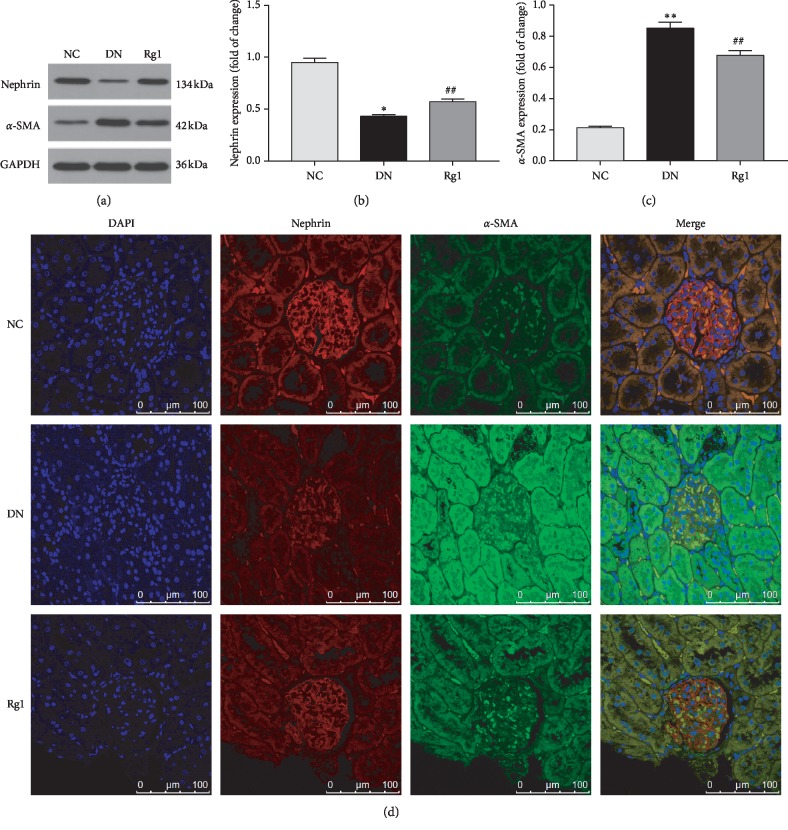
Effect of ginsenoside Rg1 on nephrin and *α*-SMA expression of kidney in SD rats: (a) representative blots for nephrin and *α*-SMA; (b, c) mean density of nephrin and *α*-SMA; (d) immunohistochemistry staining for nephrin and *α*-SMA. Data are expressed as mean ± SD, *n* = 8, ^*∗*^*P* < 0.05 and ^*∗∗*^*P* < 0.01 as compared with the NC group; ^#^*P* < 0.05 and ^##^*P* < 0.01 as compared with the DN group. Abbreviations: *α*-SMA, alpha-smooth muscle actin; EMT, epithelial-to-mesenchymal transition; DAPI, 4′, 6-diamidino-2-phenylindole.

The renal cortex of the DN rats showed a significant decrease in nephrin levels and increased *α*-SMA levels compared to controls, which was strongly indicative of EMT in the podocytes, and was reversed by Ginsenoside Rg1 (see Figures 3(a)–3(d)).

### 3.3. Ginsenoside Rg1 Regulated the AKT/GSK3 *β*/*β*-Catenin Pathway

**Figure 4:**
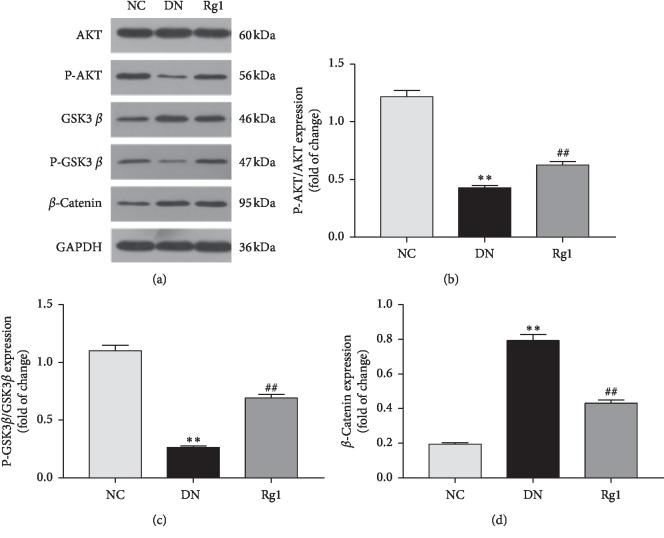
Effect of ginsenoside Rg1 on AKT/GSK3*β*/*β*-catenin pathway expression of kidney in SD rats: (a) representative blots for P-AKT, *β*-catenin, and GSK3*β*; (b–d) mean density of P-AKT, *β*-catenin, and P-GSK3*β*. Data are expressed as mean ± SD, *n* = 8, ^*∗*^*P* < 0.05 and ^*∗∗*^*P* < 0.01 as compared with the NC group;^#^*P* < 0.05 and ^##^*P* < 0.01 as compared with the DN group. Abbreviations: AKT, protein kinase B; GSK3*β*, glycogen synthase kinase 3-beta.

Since the AKT/GSK3*β*/*β*-catenin pathway is frequently implicated in EMT, we also analyzed the expression levels of its components in *in vivo* model of DN. Hyperglycemic conditions resulted in a significant increase in *β*-catenin and decrease in p-GSK3*β* and p-AKT in the podocytes and renal cortices compared to the controls, which were restored by ginsenoside Rg1 treatment (see Figures 4(a)–4(d)). Taken together, ginsenoside Rg1 inhibits EMT in the podocytes of DN rats by activating the AKT/GSK3*β*/*β*-catenin pathway.

### 3.4. Ginsenoside Rg1 Increased Autophagy in Podocytes under Hyperglycemic Conditions

**Figure 5:**
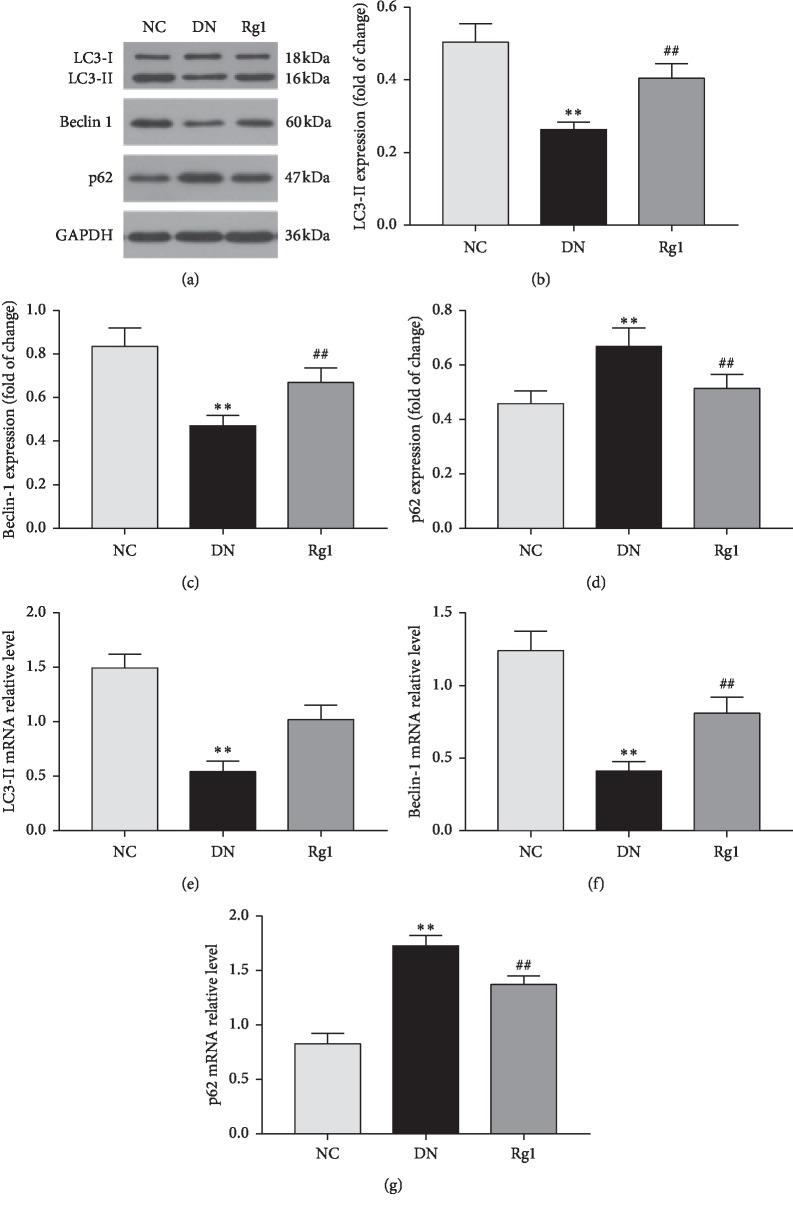
Effect of ginsenoside Rg1 on autophagy activity in SD rats: (a) western blotting results showed that ginsenoside Rg1 treatment increased the expression of beclin-1 and LC3-II in renal tissues compared to the untreated SD rats; (b–d) mean density of LC3-II, beclin-1, and p62; (e–g) RT-PCR analysis of LC3-II, beclin-1, and p62. Data are expressed as mean ± SD, *n* = 8, ^*∗*^*P* < 0.05 and ^*∗∗*^*P* < 0.01 as compared with the NC group; ^#^*P* < 0.05 and ^##^*P* < 0.01 as compared with the DN group.

**Figure 6:**
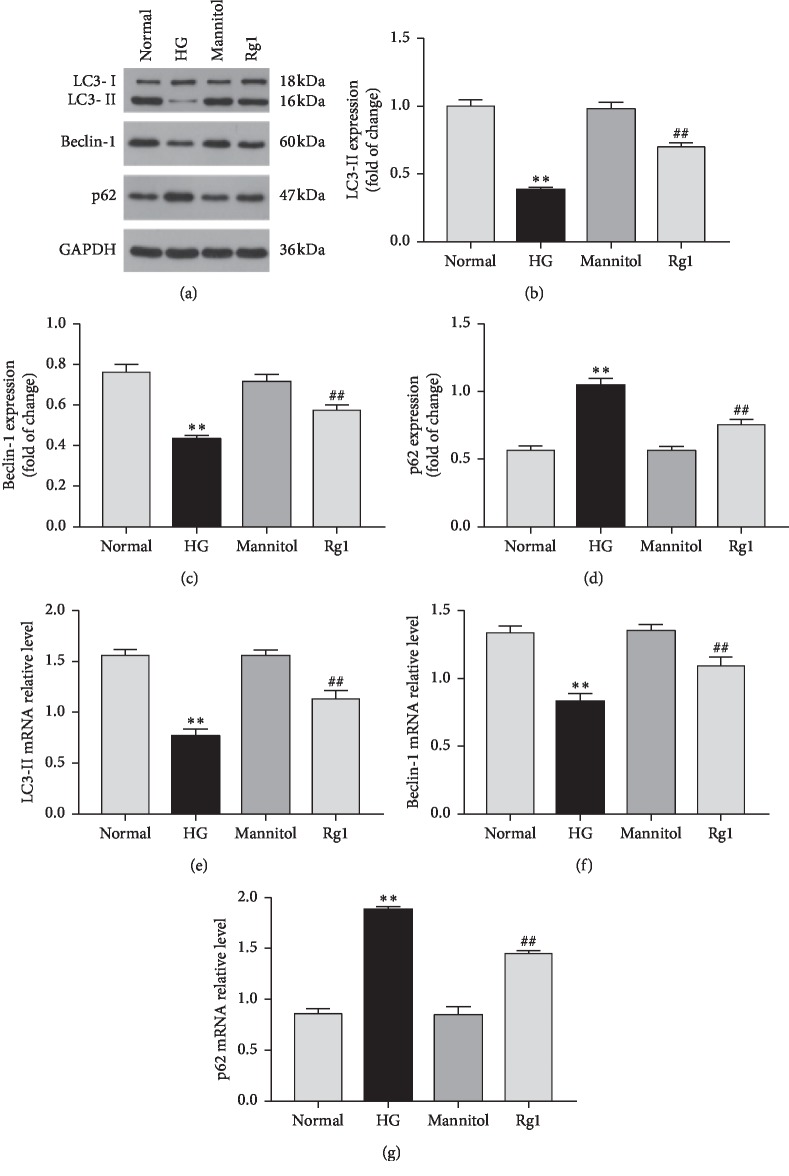
Effect of ginsenoside Rg1 on autophagy activity in podocytes under hyperglycemic conditions: (a) western blotting results showed that ginsenoside Rg1 increased the level of autophagy markers (beclin-1 and LC3 II) and decreased the level of p62 in podocytes exposed to hyperglycemia for 48 hours; (b–d) mean density of LC3-II, beclin-1, and p62; (e–g) RT-PCR analysis of LC3-II, beclin-1, and p62. Data are expressed as mean ± SD, *n* = 4, ^*∗*^*P* < 0.05 and ^*∗∗*^*P* < 0.01 as compared with the normal group; ^#^*P* < 0.05 and ^##^*P* < 0.01 as compared with the HG group. Abbreviations: HG, high glucose.

To investigate the effect of ginsenoside Rg1 on podocyte autophagy in DN, we analyzed the expression levels of LC3-II, beclin-1, and p62 in the renal cortices of the diabetic rats. Compared to the untreated DN rats, those treated with ginsenoside Rg1 showed a significant increase in the *in situ* levels of LC3-II and beclin-1 protein and mRNA, along with decreased p62 levels. Furthermore, ginsenoside Rg1 also upregulated the autophagic markers in podocytes cultured under hyperglycemic conditions. Taken together, ginsenoside Rg1 induced autophagy in the podocytes both *in vivo* (see Figures 5(a)–5(g)) and *in vitro* (see Figures 6(a)–6(g)).

### 3.5. Ginsenoside Rg1 Inhibits EMT in Podocytes by Triggering Autophagy via the AKT/GSK3*β*/*β*-Catenin Pathway

**Figure 7:**
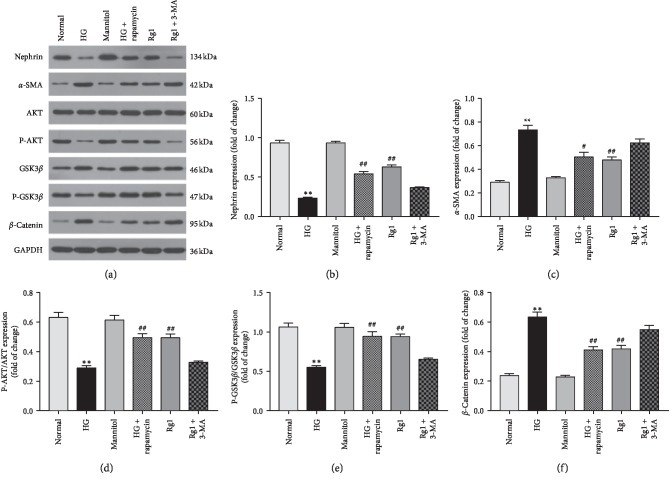
Effect of ginsenoside Rg1-induced autophagy on hyperglycemia-activated podocyte: (a) western blotting results showed both autophagy activator rapamycin, and ginsenoside Rg1 (50 *μ*M) decreased the relative *α*-SMA levels in podocyte exposed to hyperglycemia for 48 hours, increased nephrin, and activated AKT/GSK3*β*/*β*-catenin pathway, which was abolished by inhibitor 3-MA; (b–f) mean density of nephrin, *α*-SMA, P-AKT, GSK3*β*, and *β*-catenin. Data are expressed as mean ± SD, *n* = 4, ^*∗*^*P* < 0.05 and ^*∗∗*^*P* < 0.01 as compared with the normal group; ^#^*P* < 0.05 and ^##^*P* < 0.01 as compared with the HG group.

To further explore the role of autophagy in ginsenoside Rg1 action in the podocytes, we treated the cells additionally with either the autophagy activator rapamycin or inhibitor 3-MA. While ginsenoside Rg1 and rapamycin increased nephrin levels and decreased that of *α*-SMA under hyperglycemic conditions, inhibition of autophagy by 3-MA reversed the effects of ginsenoside Rg1 (see Figures 7(a)–7(c)). Furthermore, rapamycin increased p-AKT and decreased GSK3*β* activity and *β*-catenin expression along with ginsenoside Rg1, while 3-MA inhibited the AKT/GSK3*β*/*β*-catenin pathway (see Figures 7(a) and 7(d)–7(f)). Taken together, ginsenoside Rg1 inhibited EMT in the podocytes by enhancing autophagy via the activation of the AKT/GSK3*β*-catenin pathway.

## 4. Discussion

Ginsenoside Rg1 is the main active ingredient of the ginseng rhizome extract and has antiapoptotic, antioxidant, anti-inflammatory, and neuroprotective functions [17–19]. In addition, studies show that ginsenoside Rg1 can alleviate liver fibrosis, chronic obstructive pulmonary disease, and tumor invasion and migration [20–22]. Previous studies show that ginsenoside Rg1 combined with astragaloside IV can protect renal function, which may be related to antioxidant stress and inhibition of TGF-*β*1/Smads pathway [23]. In this study, we confirmed the protective effect of ginsenoside Rg1 on renal function in diabetic nephropathy rats and further examined the effects of ginsenoside Rg1 on autophagic activity and podocyte EMT.

Hyperglycemic conditions can result in abnormal renal structure and function such as tubulointerstitial fibrosis and glomerulosclerosis in the early stage of diabetes mellitus [24, 25]. The renal metabolic and pathological indices indicated significant therapeutic effects of ginsenoside Rg1 on renal fibrosis and kidney injury. Studies show that EMT in the podocytes plays the key pathological role in the development of DN by inducing cytoskeleton remodeling and fusion of the podocyte foot processes, which disrupts glomerular filtration leading to proteinuria [26]. Nephrin is the main protein in the foot process junction of the septum of the glomerular which plays an important role in preventing protein filtration [27]. *α*-SMA is a marking factor of interstitial formation as well as a marker of mesenchymal fibroblasts [28]. The expression changes of these two proteins can indirectly reflect the occurrence and development of EMT in podocytes. We found that the podocytes exposed to hyperglycemic conditions had decreased levels of the epithelial marker nephrin and increased levels of the mesenchymal marker *α*-SMA, which was reversed by ginsenoside Rg1. Therefore, the Rg1 ginsenosides suppressed hyperglycemia-induced podocyte EMT, which alleviated the DN symptoms.

Autophagy is a highly conserved eukaryotic cell cycle process, which plays an important role in cell survival and maintenance. In mammalian cells, there are three different types of autophagy: microautophagy, macroautophagy, and partner-mediated autophagy. Although the morphology of these three autophagy modes are different, the final form of autophagy is to deliver materials. The substance is transported to lysosome for degradation and recovery [29]. In recent years, there are many studies on autophagy and cancer [30, 31]. Inhibition of autophagy can lead to cell death [32], but there are few studies on the relationship between autophagy and podocyte EMT. Autophagy is downregulated in a nutrient/energy overdose state, and there is new evidence that autophagy is impaired in the glomeruli and tubules of type 1 and type 2 diabetes mellitus. In STZ-induced diabetic mice, autophagy of podocytes is inhibited, such as increased accumulation of p62/sqstm1 [33]. We also found similar observations in Wistar fat rats with type 2 diabetes [34]. Studies show that impaired autophagy can lead to podocyte injury and proteinuria, whereas restoring autophagy repairs damaged podocytes [35,36]. In this study, hyperglycemic conditions both in vivo and in vitro decreased LC3-II and beclin-1 and increased P62 levels, indicating inhibition of autophagy. After the autophagy-activating drugs were used to intervene in podocytes, the expression of the podoccyte marker protein nephrin was increased and the expression of the mesenchymal marker *α*-SMA was decreased, indicating that autophagy can protect podocyte injury. Treatment with ginsenoside Rg1 restored autophagy in the podocytes, which is the likely mechanism underlying inhibition of EMT. The autophagy inhibitor 3-MA abolished the protective effects of ginsenoside Rg1, thereby validating our hypothesis.

Podocyte EMT involves various signaling cascades, such as Wnt and TGF-*β* [37]. Hyperglycemia can be used as an inducer to induce EMT in podocytes through Wnt/*β*-catenin pathway [38]. Increased expression of *β*-catenin in diabetic patients and mouse models plays a key role in inducing podocyte EMT [39]. Therefore, the effective regulation of the Wnt/*β*-catenin pathway may be a key node in the treatment of diabetic nephropathy. Glycogen synthase kinase 3*β* is a widely expressed serine/threonine protein kinase, as an important substrate of AKT, originally identified as an enzyme needed to regulate glycogen metabolism, and is now found to be a multifunctional kinase that plays an important role in many cell processes and diseases [40–42]. GSK3*β* is negatively regulated by AKT, which inhibits glycogen synthesis and lowers blood glucose by inhibiting GSK3*β* phosphorylation [43]. In addition, GSK3*β* plays an important role in cell growth and development by phosphorylating a variety of endogenous substrates. Decreased activity of AKT could inhibit the phosphorylation of GSK3*β* and then leads to the accumulation of *β*-catenin in the cytoplasm of podocytes. *β*-catenin eventually enters the nucleus and triggers EMT [44]. Astragaloside IV inhibits EMT by targeting the AKT/GSK3*β*/*β*-catenin pathway, which weakens the invasion and migration of hepatocellular carcinoma cells [45]. Autophagy is an important part of cellular activity; however, its relationship with the AKT/GSK3*β*/*β*-catenin pathway is still unclear. According to the results of this study, the activation of autophagy in hyperglycemic conditions will promote Akt phosphorylation and then activate the phosphorylation of GSK3*β,* which is the key protein of Wnt/*β*-catenin pathway. This will lead to the decrease in accumulation of *β*-catenin and expression of *α*-SMA and ultimately alleviate podocyte EMT. The amelioration of podocyte EMT after ginsenoside Rg1 intervention may be correlated to the regulation of the AKT/GSK3*β*/*β*-catenin pathway by ginsenoside Rg1-enhanced autophagic activity.

In conclusion, ginsenoside Rg1 inhibits podocyte EMT by inducing autophagy via regulation of the AKT/GSK3*β*/*β*-catenin pathway, providing a reasonable basis for its therapeutic use in DN.

## 5. Conclusions

The data obtained in the present study indicate that ginsenoside Rg1 induces autophagy in renal tissues and podocytes exposed to high glucose via activation of the AKT/GSK3*β*/*β*-catenin pathway. Meanwhile, ginsenoside Rg1 improves albuminuria and the renal function in DN rats. These findings point to the possible therapeutic use of ginsenoside Rg1 in DN. However, additional studies are needed to comprehensively understand the role of ginsenoside Rg1 in ameliorating DN.

## Figures and Tables

**Table 1:** RT fluorescence quantitative PCR primers.

Genes	Forward primer 5′-3′	Reverse primer 5′-3′
LC3-II	ATCAACATTCTGACGGAGCGG	ATCTGCCTGCTTGTCCTGGTT
p62	TGGACCACAAGGAAATACAATCA	CCTCCTTGGCTTTGTCTCTCATC
Beclin-1	GAATGGAGGGGTCTAAGGCG	CTTCCTCCTGGCTCTCTCCT
*β*-Catenin	ATTACGACAGACTGCCTTCAGATC	GAGCAGACAGACAGCACCTTCA

## Data Availability

The data used to support the findings of this study are included within the article.
